# Mechanism of Hip Arthropathy in Ankylosing Spondylitis: Abnormal Myeloperoxidase and Phagosome

**DOI:** 10.3389/fimmu.2021.572592

**Published:** 2021-11-22

**Authors:** Chaojie Yu, Xinli Zhan, Tuo Liang, Liyi Chen, Zide Zhang, Jie Jiang, Jiang Xue, Jiarui Chen, Chong Liu

**Affiliations:** Spine and Osteopathy Ward, The First Affiliated Hospital of Guangxi Medical University, Nanning, China

**Keywords:** ankylosing spondylitis, proteomics, bioinformatics, myeloperoxidase, phagosome

## Abstract

**Background:**

The pathogenesis of Ankylosing spondylitis (AS) has not been elucidated, especially involving hip joint disease. The purpose of this study was to analyze the proteome of diseased hip in AS and to identify key protein biomarkers.

**Material and Methods:**

We used label-free quantification combined with liquid chromatography mass spectrometry (LC–MS/MS) to screen for differentially expressed proteins in hip ligament samples between AS and No-AS groups. Key protein was screened by Bioinformatics methods. and verified by *in vitro* experiments.

**Results:**

There were 3,755 identified proteins, of which 92.916% were quantified. A total of 193 DEPs (49 upregulated proteins and 144 downregulated proteins) were identified according to P < 0.01 and Log|FC| > 1. DEPs were mainly involved in cell compartment, including the vacuolar lumen, azurophil granule, primary lysosome, etc. The main KEGG pathway included Phagosome, Glycerophospholipid metabolism, Lysine degradation, Pentose phosphate pathway. Myeloperoxidase (MPO) was identified as a key protein involved in Phagosome pathway. The experiment of siRNA interfering with cells further confirmed that the upregulated MPO may promote the inflammatory response of fibroblasts.

**Conclusions:**

The overexpression of MPO may contribute to the autoimmune inflammatory response of AS-affected hip joint through the phagosome pathway.

## 1 Introduction

Ankylosing spondylitis (AS) is a chronic systemic inflammatory disease with an incidence of 0.1~1.4% (1). This disease mainly occurs in young males and is mainly characterized by spinal arthritis and sacroiliac arthritis, and associated with a number of other features including peripheral arthritis, psoriasis, anterior uveitis, inflammatory bowel disease, etc. (1–5). Main pathological structures of arthritis are cartilage junctions and attachment points (6, 7). Due to the slow progression, it usually takes 5 to 10 years after the onset of symptoms to be diagnosed. In the later stages, loss of activity of spinal and sacroiliac joint results in poor quality of life, increased financial burden, and more serious mental illness (6, 8, 9). There are no effective radical drugs at present. Previous researches focused on the overall pathogenesis of AS having involved multiple fields: genetics, intestinal diseases, intracellular environmental barriers, hormones, etc. (1, 2, 4, 10, 11). Multiple genes abnormally express proteins and involve different pathways in different diseased tissues, which may be the main factor leading to AS. However, it is complex. The overall pathogenesis of AS has not been fully elucidated. In particular, there are few reports on the mechanism of hip arthropathy in patients with AS.

Proteins are the end products of gene expression and can better and directly illuminate the processes behind the manifestation of diseases. Comparative proteomics methods are widely used to analyze the relative amounts of proteins in two or more biological samples to screen for biomarker proteins for judgment and prognosis (12–14). Label-free quantitative (LFQ) analysis is one of proteomics methods (12, 13). Compared to label quantitative analysis, LFQ can avoid the drawbacks of labeling quantification techniques: additional sample processing, sample complexity caused by incomplete labeling, difficulty in detecting low abundance peptides, limited number of labeled samples, etc. (13, 15).

In this study, LFQ combined with bioinformatics technology was used to screen potential biomarkers. It was found that the protein changes in hip joint during the development of AS to speculate the signal transduction pathway. This provides a preliminary insight into the molecular characteristics of hip arthropathy in patients with AS, which is conducive to a further comprehensive understanding of the biological characteristics of AS and to provide new molecular targets for personalized treatment.

## 2 Material and Methods

### 2.1 Patients and Samples Collection

From December 2018 to August 2019, a total of 12 ligament samples were collected from patients undergoing hip replacement in the First Affiliated Hospital of Guangxi Medical University. The study population included the AS group (n = 6) and No-AS group (n = 6). The AS group included patients with AS combined with hip arthropathy. The No-AS group included six patients with aseptic femoral head necrosis (AFHN).

Inclusion criteria: (1) patients with AS combined with hip arthropathy or patients with AFHN; (2) hip replacement. Exclusion criteria: (1) combined with other connective tissue diseases such as rheumatoid, systemic lupus erythematosus; (2) combined with tumor, trauma, and other diseases. All tissues collected during the operation were cleaned with phosphate buffered solution (PBS, Solarbio, Beijing, China) and put into the labeled cryopreservation tubes, respectively. Cryopreservation tubes were stored in a −80°C refrigerator until they were used.

General data [age, gender, body mass index (BMI), etc.] and preoperative examination data [erythrocyte sedimentation rate (ESR), blood routine, human leukocyte antigen (HLA)-B27, etc.] were collected. The diagnosis of AS was based on the revised New York standard in 1984 (16). The diagnosis of AFHN was based on a pathological examination of femoral head (17). We performed label-free protein analysis with the assistance of KangChen Bio-tech (Shanghai, China). All procedures were performed in compliance with the resolution of Helsinki and approved by the Local Ethics Committee. All participants received written informed consent.

### 2.2 Protein Preparation and Label-Free Quantification

#### 2.2.1 Sample Lysis

The RIPA mixture is prepared immediately before use and placed on ice to cool. It is composed of RIPA lysis buffer (modified, Kangchen Bio-tech, Shanghai, China), Protease inhibitor cocktail (Kangchen Bio-tech, Shanghai, China), and 1 mM PMSF (Phenylmethylsulfonyl fluoride) (Sigma-Aldrich, St. Louis, MO, USA). About 100 mg sample tissue (1×10*7 cells) was thoroughly mixed with 1,000 μl RIPA mixture, which was homogenized and sonicated to dissolve at 4°C for approximately 5 min. After centrifugation (speed 14,000 g, 15 min) at 4°C, the supernatant was transferred to the new EP tube and placed on ice.

#### 2.2.2 BCA Assay

According to the instructions of the BCA (Bicin-choninic Acid) Protein Assay Kit (Kangchen Bio-tech, Shanghai, China), reagent A and reagent B were mixed at A 50:1 ratio and added to a 96-well plate (160 µl/per well, five wells for calibration curve and one well for blank). Then 20 µl sample (dilute 5~10 times) or calibration standard protein (five different concentration levels) was respectively added into the well. The plates were shaken and incubated at 37°C for 30 min and were read with 562 nm wavelength. Protein concentration of each sample was calculated according to the calibration curve.

#### 2.2.3 Acetone Precipitation

For each sample, 100 µg protein was taken into an EP tube and diluted to 1 mg/ml by RIPA buffer. Then 4~6-fold volumes of pre-chilled acetone (−20°C, Sangon Biotech, Shanghai, China) was added into the EP tube (alkylated protein), which was shaken on ice for 30 min or incubated at −20°C overnight. After centrifugation (speed 10,000 g, 4°C), the supernatant was carefully discarded without disturbing the pellet. The sample was washed twice with 200 µl 80% chilled acetone.

#### 2.2.4 Resuspend Protein for Tryptic Digest

Two hundred µl 1% SDC (sodium deoxycholate, Sigma-Aldrich, St. Louis, MO, USA) and 100 mM ABC (ammonium bicarbonate) (Sigma-Aldrich, St. Louis, MO, USA) was added into the EP tube, mixed with vortex, and spun down. The EP tube was sonicated for 5~30 min in water bath to dissolve protein. Five mmol TCEP (tris 2-carboxyethyl phosphine) (Sigma-Aldrich, St. Louis, MO, USA) was added into the EP tube (sample protein), which was incubated and mixed at 55°C for 10 min. Ten mmol IAA (iodoacetamide) (Sigma-Aldrich, St. Louis, MO, USA) was added after samples were cooled down to room temperature (RT). The EP tube was incubated in the dark for 15 min. Trypsin (sequence grade) (Promega, Madison, WI, USA) was resuspended with resuspension buffer to 0.5 µg/µl and incubated at RT for 5 min. Trypsin solution (protein:trypsin = 50:1) was added into the EP tube. They were mixed well and spun down, then incubated at 37°C with thermomixer for about 8 h or overnight.

#### 2.2.5 Cleaning Up of SDC

SDC was precipitated after 2% TFA (Trifluoroacetic Acid, HPLC) (Sigma-Aldrich, St. Louis, MO, USA) added into the EP tube. After centrifugation at top speed, the supernatant was transferred to a new EP tube. N * 100 µl 2% TFA was added into the pellet to extract co-precipitated peptides. The step was repeated twice. Three supernatants were merged. After centrifugation at top speed for 10–20 min, the supernatant was carefully transferred to a new EP tube, leaving peptide samples.

#### 2.2.6 Peptide Desalting for Base-RP Fractionation

Buffer A [0.1% FA (formic acid) (LC-MS, Sigma-Aldrich, St. Louis, MO, USA), H2O, 2% ACN (Acetonitrile) (LC-MS, J.T.Baker, PA, USA)] and Buffer B (0.1% FA, 70% ACN) were prepared. C18 (3M) (Sigma-Aldrich, St. Louis, MO, USA) column was equilibrated with added 500 µl ACN. ACN was washed out with 500 µl 0.1% FA two times. The peptide solution was added into the C18 column. After centrifugation at low speed, liquid (A) was collected. The steps were repeated again. Peptide was eluted with 400 µl 70% ACN, and liquid (A) was collected. The desalting step (Equilibrate to Elute) was repeated by liquid (A) once again. Two liquids were merged and dried by vacuum under 4°C or RT. Buffer A was added to redissolve the polypeptide to 1 buffer g/buffer L for LC-MS/MS detection or −80°C storage.

#### 2.2.7 Separation *via* Nano-UPLC

For each sample, 2 µg peptide were separated and detected with a nano-UPLC (EASY-nLC1200, Thermo Scientific, MA, USA) coupled to Q-Exactive mass spectrometry (Thermo Scientific, MA, USA). Analysis was performed using a reversed-phase column (100 µm, ID × 15 cm, Reprosil-Pur 120 C18-AQ, 1.9µm, Dr. Math). The mobile phases solution includes phase A solution (0.1% FA, 2% ACN) and phase B solution (80% ACN, 0.1% FA). The chromatographic column is balanced by 100% phase A solution. Through an automatic sampler, the samples were directly delivered to the chromatographic column to be separated at a flow rate of 300 nl/min and a gradient of 120 min. Phase B solution is used in sequence: 8 to 30% for 92 min, 30 to 40% for 20 min, 40 to 100% for 2 min, 100% for 2 min, 100 to 2% for 2 min, and 2% for 2 min.

#### 2.2.8 LC-MS/MS

Data dependent acquisition was performed in profile and positive mode with Orbitrap analyzer at a resolution of 70,000 (200m/z) and m/z range of 350–1,600 for MS1. For MS2, the resolution was set to 17,500 with a dynamic first mass. The automatic gain control (AGC) target for MS1 was set to 3.0 E*6 with max IT 50 ms, and 5.0 E*4 for MS2 with max IT 100 ms. The top 20 most intense ions were fragmented by HCD with normalized collision energy (NCE) of 27%, and isolation window of 2 m/z. The dynamic exclusion time window was 30 s.

#### 2.2.9 MaxQuant Database Search

Raw MS files were processed with MaxQuant (Version 1.5.6.0). The protein sequence database (Uniprot_organism_2016_09) was downloaded from UNIPROT. This database and its reverse decoy were then searched against by MaxQuant software. The quantification type was LFQ with match between run and intensity-based absolute quantification (iBAQ). Trypsin was set as specific enzyme with up to 3 miss cleavage. Oxidation (M) and Acetyl (protein N-term) were considered as variable modification (max number of modifications per peptide is 3), Carbamidomethyl (C) was set as fixed modification. Both peptide and protein of false discovery rate (FDR) should be less than 0.01. Only unique and razor peptides were used for quantification. All the other parameters were reserved as default.

### 2.3 Bioinformatics Analysis

#### 2.3.1 Differentially Expressed Proteins

Differentially expressed proteins (DEPs) were defined as P < 0.01, the ratio of | AS group/No-AS group | > 2 or |Log Fold change (FC) |>1, unique peptide > = 2 protein. Relative protein expression values between two groups were compared. There were six biological replications in this experiment, which were tested by the t-test. False Discovery Rate (FDR) <0.05.

#### 2.3.2 GO and KEGG Enrichment Analysis

DEPs were analyzed for GO (Gene Ontology) and KEGG (Kyoto Encyclopedia of Genes and Genomes) enrichment by the plugin ClueGO (18) in the Cytoscape software (version: 3.6.1) (19). There are three categories of GO annotation: Biological process (BP), Cell compartment (CC), and Molecular function (MF). The standard setting with statistically significant difference was P < 0.05, and Kappa score was 0.4. Bonferroni corrected P < 0.05 is considered to be valid.

#### 2.3.3 PPI Network Construction

We integrated the DEPs into PPI network by String (20) (version 11.0) to evaluate the interaction between DEPs. A composite score >0.4 was considered to be a statistically significant interaction. Analysis data of the PPI network were loaded into Cytoscape software for visual adjustment.

#### 2.3.4 Identification of Key Protein

Top 30 hub proteins were screened by the plug-in CytoHubba (21) in Cytoscape software from PPI networks. Key protein was selected from the correlations of hub proteins and KEGG pathways.

### 2.4 Cell Culture and Transfection

Fibroblasts from the two groups were separated and cultured. The fibroblasts were cultured in specific medium DMEM (10% FBS, 100 μg/ml streptomycin and 100 IU/ml penicillin) at 37°C in a 5% CO2 incubator. Cells in the AS group were transfected with Lipofectamine 3000 (L3000015, Thermofisher Scientific, MA, USA) according to the manufacturer’s instructions. The siRNA-MPO-treated cells were siRNA-MPO group. As a negative control, cells treated with the siRNA-NC group were siRNA-NC group. The transfection efficiency was evaluated by Western blotting and quantitative real-time PCR (QPCR).

### 2.5 Western Blotting Analysis

Western blot analysis was performed between groups. Total proteins were extracted from cell samples. BCA protein concentration determination kit (Servicebio, Wuhan, China) was used to determine the total protein concentration. The proteins were separated by 10% SDS-PAGE (Servicebio, Wuhan, China) and transferred to cellulose nitrate membrane. The membrane was sealed in 5% degreased dry milk in 37°C TBS buffer (Servicebio, Wuhan, China) for 1 h and incubated overnight with primary antibodies (MPO antibodies, Abcam, Europe) at 4°C. Then, the membranes were washed three times in TBST and incubated with secondary antibodies at room temperature for 30 min. A scanner (EPSON V300, Japan) was used to sweep membrane and upload band data. Adobe PhotoShop (Adobe, USA) software was used to process the color, and Alpha Innotech (Alpha Innotech, USA) software was used to analyze the gray values of band. Relative expression level of protein = (gray value of the target protein band)/(gray value of the GAPHD protein band).

### 2.6 Quantitative Real-Time PCR

According to the manufacturer’s instructions, Tripure reagent (Roche, Switzerland) was applied to extract total RNA from cells. The cDNA was synthesized according to the reaction system. Next, quantitative real-time PCR(QPCR)was performed using a 2×UltraSYBR Mixture. The qPCR condition was set as follows: 95°C 10 min, 95°C 15 s, 60°C 20 s, 72°C 25 s, 40 cycles, followed by a 5-min final extension at 72°C. The 2-ΔΔCt method (Ct of target genes minus the Ct of GAPDH) (22) was used to calculate the relative expression levels of mRNA. The primer sequence of MPO is as follows: TCGGTACCCAGTTCAGGAAG (forward) and CCAGGTTCAATGCAGGAAGT (reverse).

### 2.7 Enzyme-Linked Immunosorbent Assay

Enzyme-linked immunosorbent assay (ELISA) was used to detect inflammatory markers in cells before and after transfection. The concentrations of interleukin 6 (IL-6), interleukin 8 (IL-8), and tumor necrosis factor α (TNF-α) in the cell culture medium were determined by ELISA sandwich method according to the instructions of ELISA assay kit (MLBIO, Shanghai, China). The collected cell culture medium was centrifuged at 4°C for 10 min at 1,000 RPM to obtain the supernatant. The absorbance value (OD value) of each culture medium was determined at 450 nm using a microplate analyzer (Mlbio, Shanghai, China). Finally, the concentrations of IL-6, IL8, and TNF-α in each sample were obtained through the standard curve equation.

### 2.8 Statistical Analysis

The data were expressed as the mean ± SD, and differences between two groups were analyzed with Student’s t-test. P < 0.05 was considered statistically significant. Bonferroni or Benjamini-Hochberg adjusted FDR, which was <0.05. Statistical analysis was performed using the Statistical Program for Social Sciences (SPSS) software 25.0 (IBM, USA).

## 3 Results

### 3.1 Clinical Data of Patients

**Figure 1:**
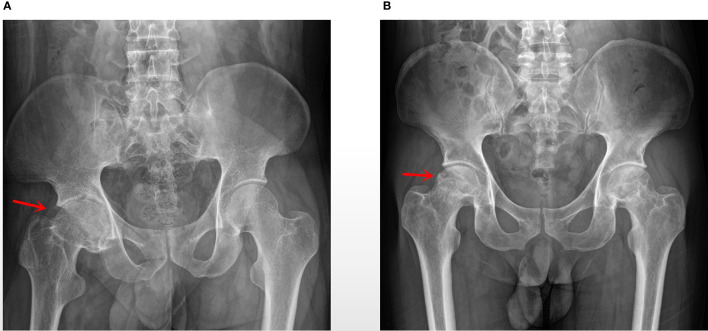
**(A)** AS group: a patient with AS, 28 years old, male, BMI: 15.34, ESR: 41 mm/h, HLA-B27 (+); **(B)** No-AS group: a patient with aseptic femoral head necrosis, 43 years old, male, BMI: 24.564, ESR: 63 mm/h.

In this study, ligament tissues from 12 patients undergoing hip arthroplasty were collected and divided into AS (n=6) and NO-AS groups (n=6) (**Figure 1** and **Table 1**). Except for HLA-B27, there were statistically significant differences in age and gender between two groups. The characteristics of AS patients involving hip arthropathy were younger and HLA-B27 positive.

**Table 1:** Clinical information of patients of the two groups.

	AS group n = 6	No-AS group n = 6	P value
Age (year)	34.167 ± 10.362	48.333 ± 11.272	0.050*
Gender (M / F)	3 / 3	3 / 3	
BMI (kg/m^2^)	22.692 ± 10.286	21.725 ± 4.141	0.835
ESR (mm/h)	58.000 ± 25.581	53.667 ± 31.910	0.808
Neutrophils in blood (*10^9/L)	5.708 ± 1.240	7.045 ± 3.994	0.452
Lymphocyte in blood (*10^9/L)	1.865 ± 0.882	1.443 ± 0.406	0.323
HLA-B27 (+)	6	0	

*Compared with Easy group, P < 0.05.

### 3.2 Differentially Expressed Proteins

**Figure 2:**
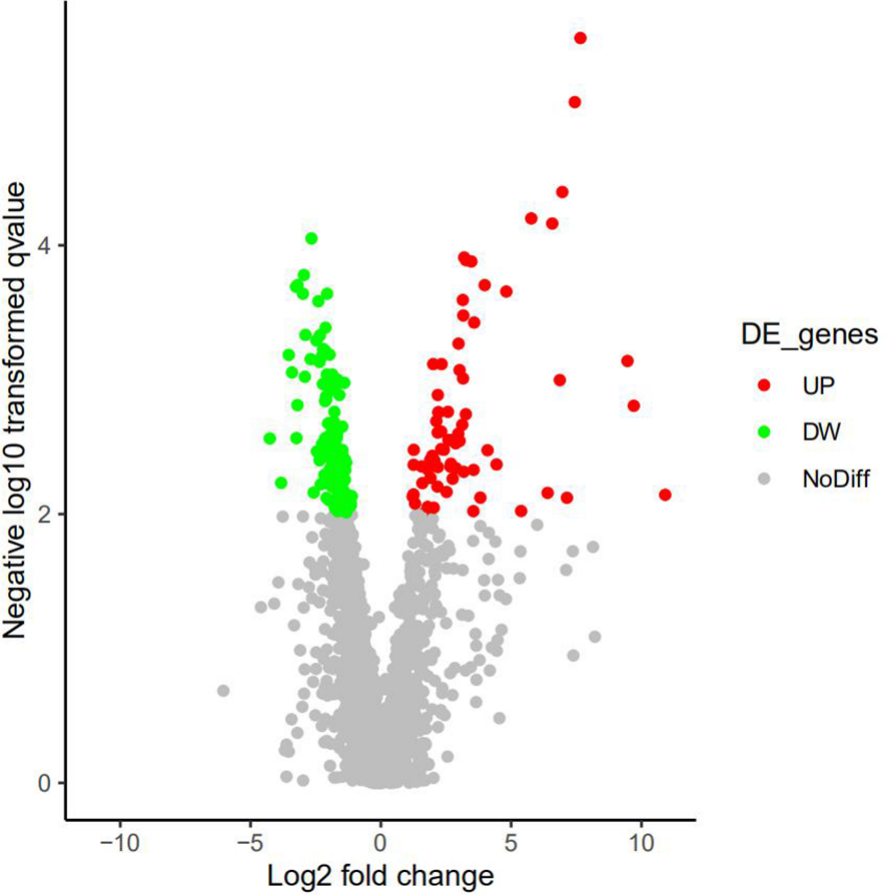
Active volcano map of proteins, screening criteria of DEPs: P.value<0.05 and Log|logFC|>1. Red dots represent upregulated proteins, and the green dots represent downregulated proteins. Gray dots mean these proteins do not satisfy the screening criteria. X-axis represents Log2|FC|, and the y-axis represents -Log10|P-value|.

**Figure 3:**
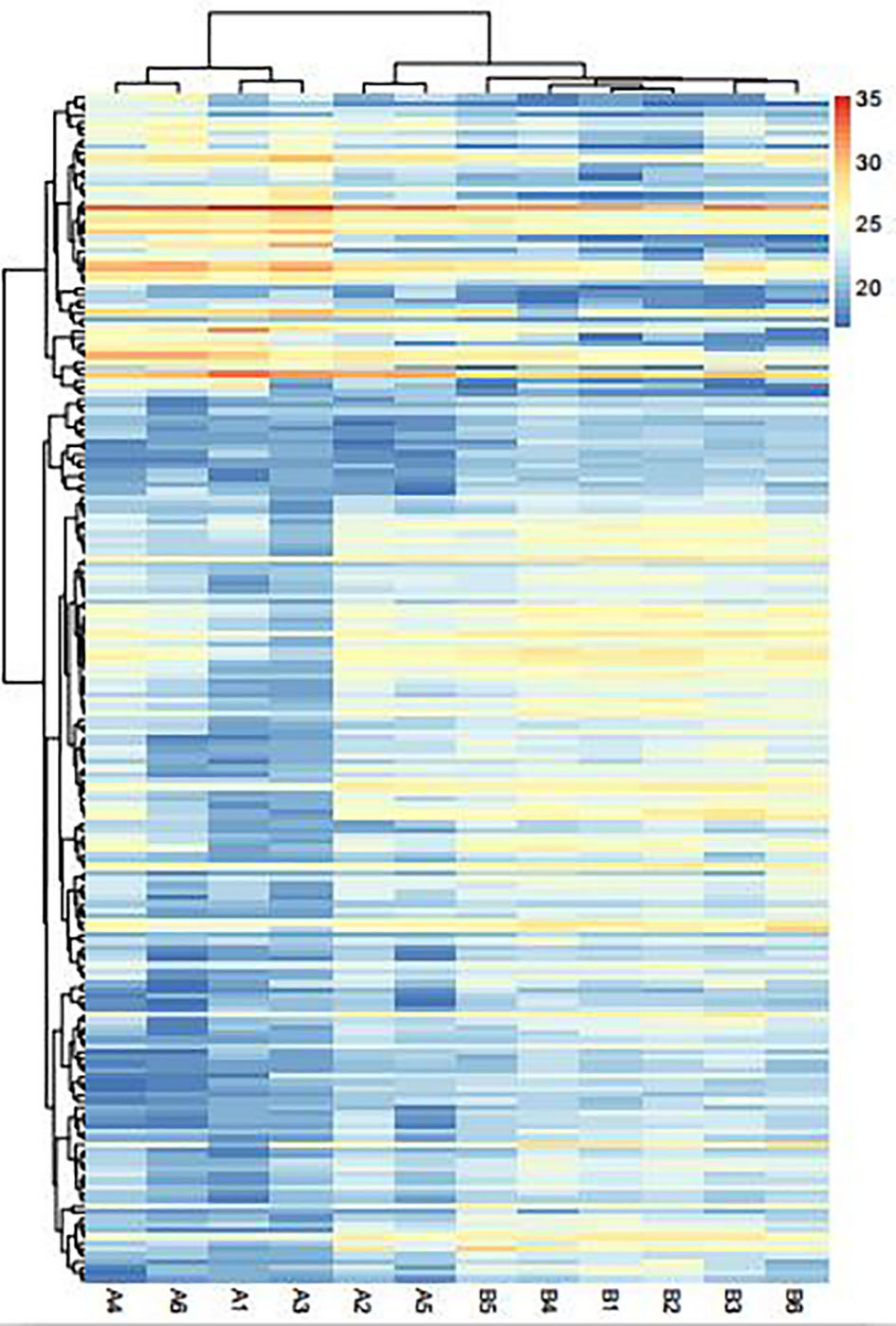
Heat map of the DEPs between AS **(A1–A6)** and No-AS **(B1–B6)** groups. Red areas are the upregulated proteins, and the blue areas are the downregulated proteins.

A total of 3,755 proteins were identified, among which 3,489 proteins had quantitative information. There were 193 DEPs (49 upregulated proteins and 14 downregulated proteins) between AS and No-AS groups (P.value < 0.05) (**Table 2**). Both volcano map and heat map showed significant differences in proteins between two groups (**Figures 2**, **3**).

**Table 2:** Statistical results of polypeptides and proteins.

	Count
Total polypeptide	36,639
Total protein	3,755
Quantifiable protein	3,489
Differentially expressed proteins (DEPs)	193

### 3.3 GO Functional Enrichment Analysis

**Figure 4:**
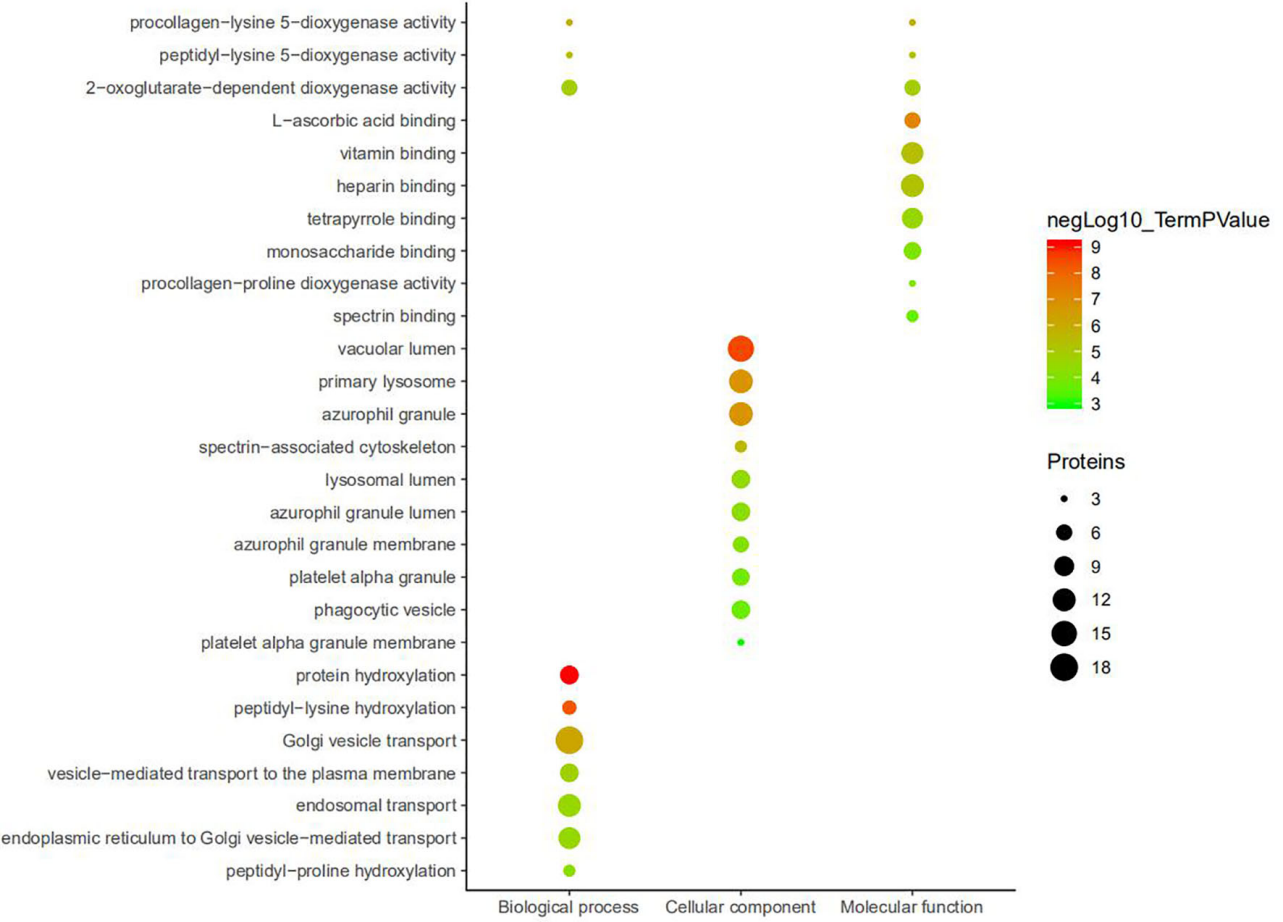
Top 10 GO enrichment analysis of DEPs. Function of the DEGs in tissues is described according to its GO characteristics (Biological process, Molecular function, Cell components). -Log10 (p. adj.value) < 0.05 and count of gene is > = 3.

DEPs were subjected to GO enrichment analysis, and items were screened according to P.adj.value (P.adj.value<0.05). It was found that there were a large number of DEPs enriched in BP, CC, and MF. Among them, the DEPs enriched in BP and CC are slightly more than those enriched in MF. In BP, most proteins were mainly involved in Golgi vesicle transport, endosomal transport, endoplasmic reticulum to Golgi vesicle–mediated transport, etc. In terms of CC, most proteins were involved in vacuolar lumen, azurophil granule, primary lysosome, etc. In MF, most proteins were involved in vitamin binding, heparin binding, tetrapyrrole binding, etc. The enrichment results of DEPS showed common characteristics between BP and CC, including procollagen-lysine 5-dioxygenase activity, peptidyl-lysine 5-dioxygenase activity, 2-oxoglutarate-dependent dioxygenase activity (**Figure 4**).

### 3.4 KEGG Pathway Enrichment Analysis

**Figure 5:**
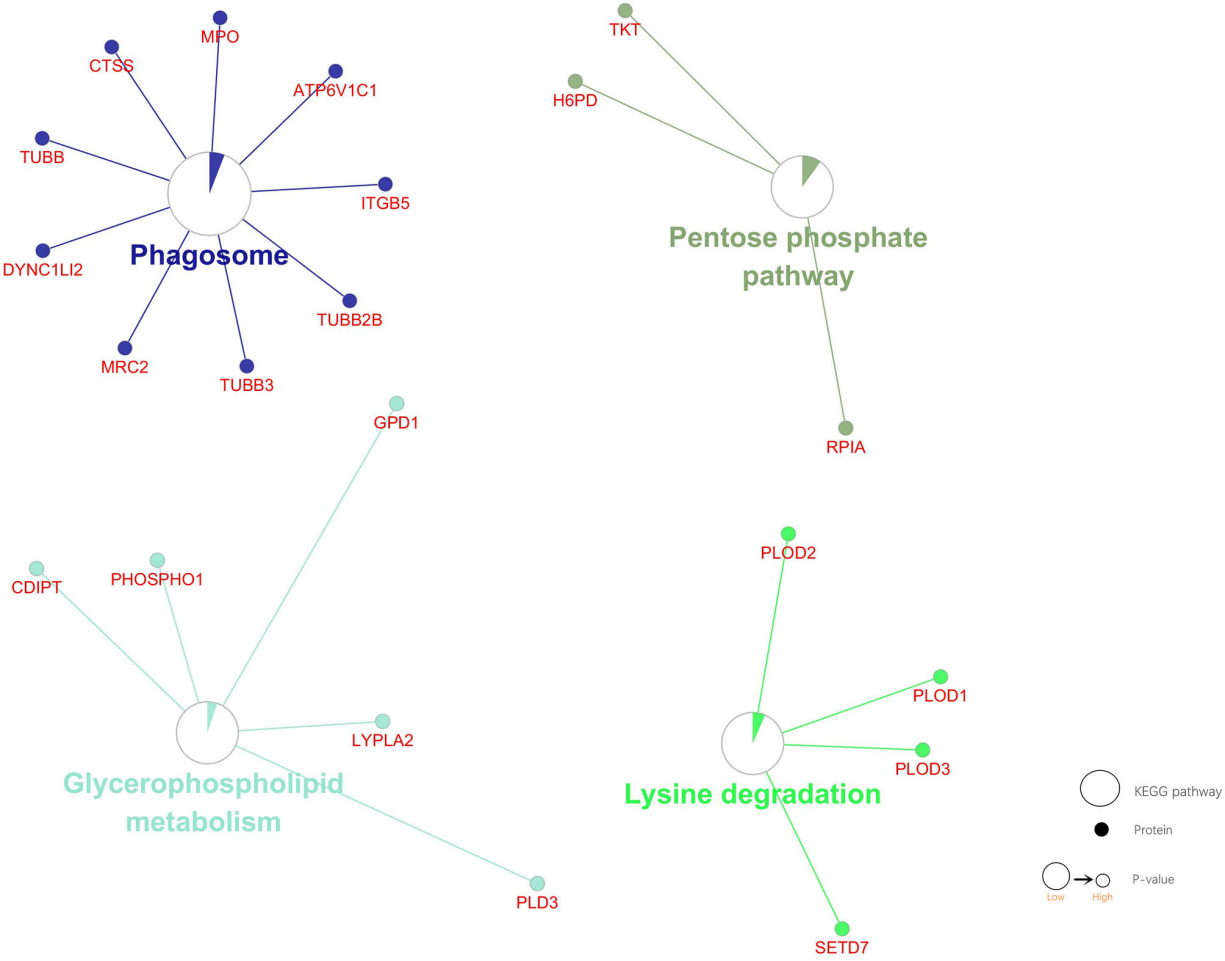
KEGG pathway enrichment analysis of DEPs. Four pathways and associated DEPs are shown. Dot: protein. Circulation: KEGG pathway. Circle size: the smaller the P value, the bigger the circle. Area of the color in circle represents the percentage of associated DEPs in the Circle proteins.

KEGG pathway enrichment analysis of DEPs was performed by the Gluego. Four pathways were identified, including Phagosome, Glycerophospholipid metabolism, Lysine degradation, Pentose phosphate pathway (P. adj. value<0.05). Nine proteins were involved in the Phagosome. Three proteins were involved in the Pentose phosphate pathway. Five proteins were involved in the Glycerophospholipid metabolism. Four proteins were involved in the Lysine degradation. The P value of the Phagosome pathway is the smallest, and the number of DEPs involved in it is the largest. The number of DEPs involved in the Pentose phosphate pathway is the lowest. These DEPs are involved in the abnormal operation of the Phagosome, which may be the most important pathogenetic pathway. And key proteins may play important roles in this pathway (**Figure 5**).

### 3.5 PPI Network Construction

**Figure 6:**
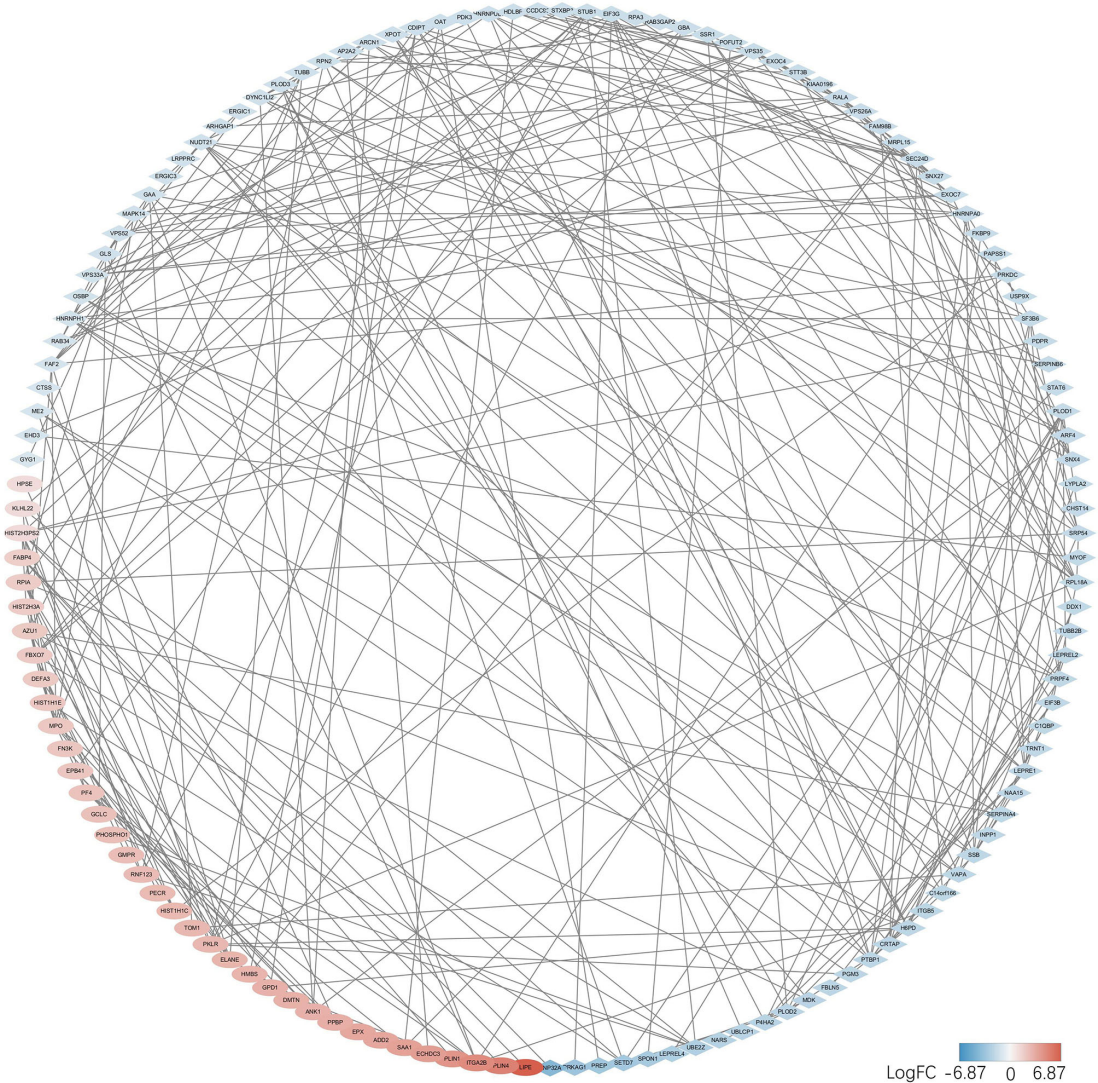
DEPs in the PPI networks are visualized by the Cytoscape. Red balls represent the upregulated proteins, and the blue diamonds represent the downregulated proteins.

String database was used to evaluate the interaction between two groups of DEPs, and PPI network was constructed. Data were entered into the Cytoscape for visual adjustment. DEPs visual network has 188 nodes and 332 edges (**Figure 6**). As can be seen from the figure, the number of downregulated DEPs was higher than that of upregulated DEPs. However, the absolute value of FC in the upregulated DEPs seemed to be generally larger than that in the downregulated DEPs. DEPs, whether upregulated or downregulated, are closely linked to each other and together constitute a complex protein-protein interaction network.

### 3.6 Identification of Key Proteins

**Figure 7:**
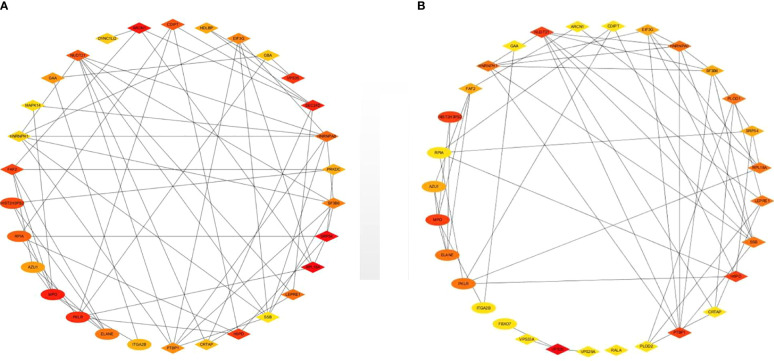
Top 30 hub proteins were obtained respectively by Degree and Closeness method in CytoHubba. **(A)** Hub proteins in Degree method, **(B)** hub proteins in Closeness method.

**Figure 8:**
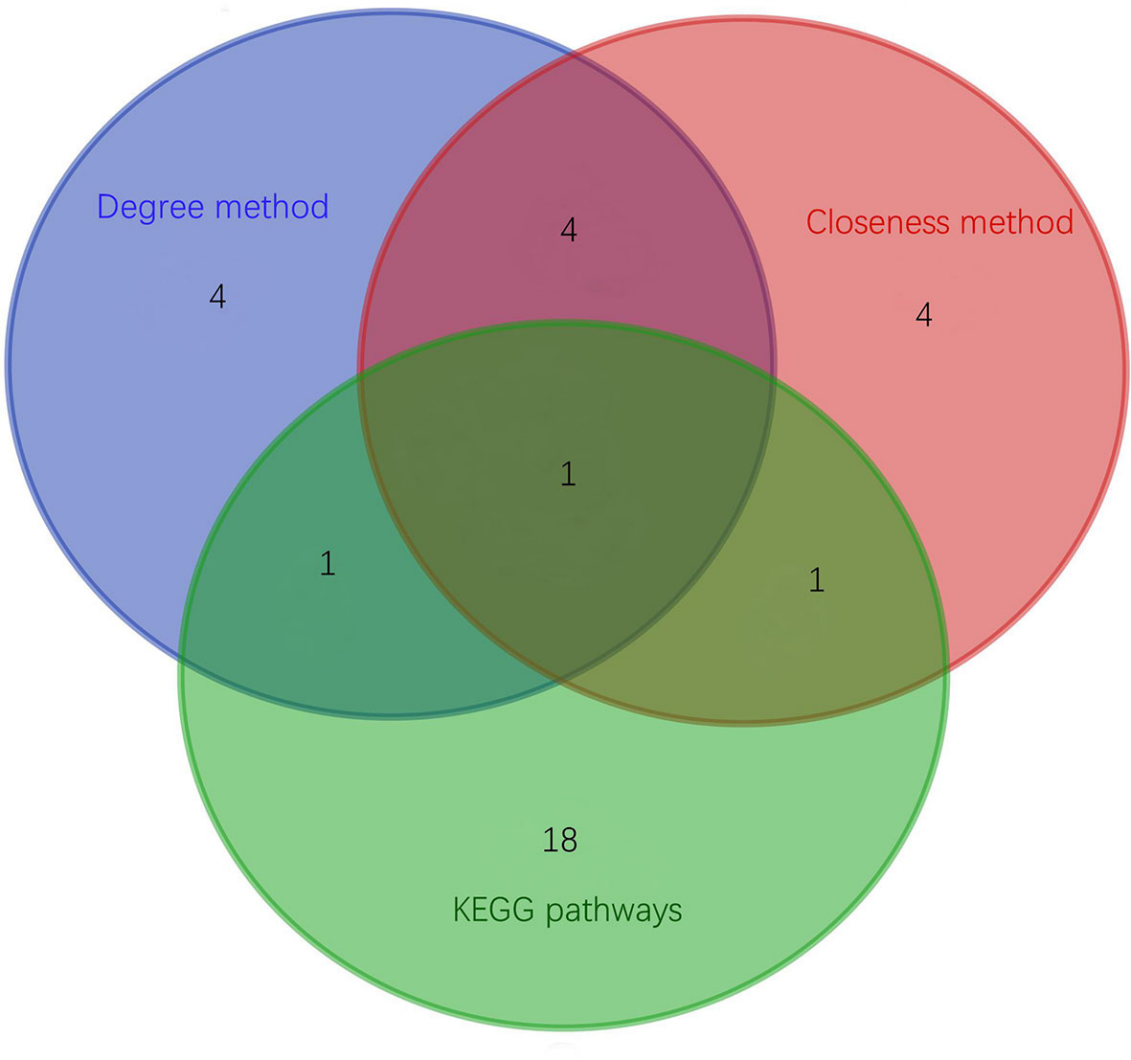
Proteins intersection map of KEGG pathways, Degree method and Closeness method.

**Figure 9:**
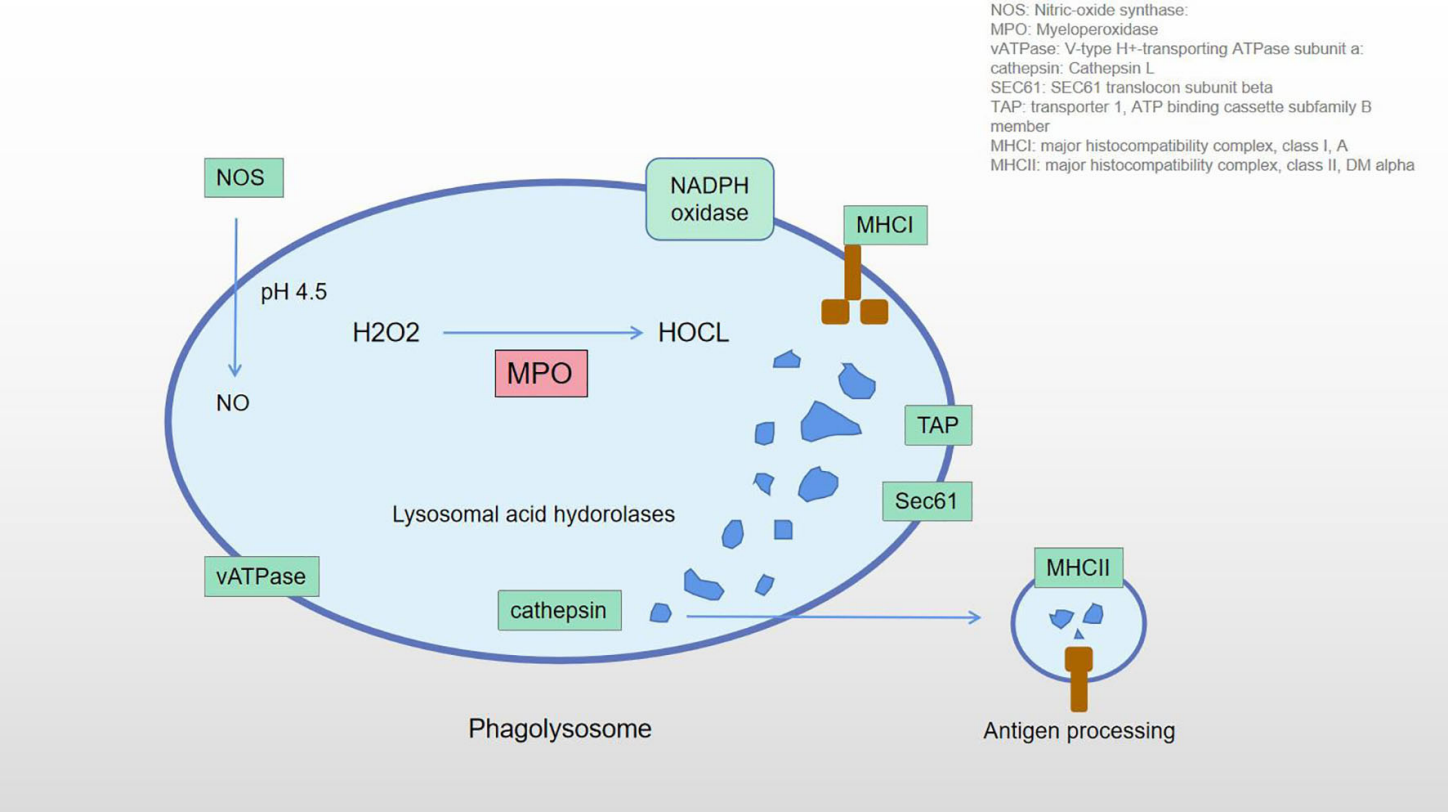
The role of MPO in phagolysosome. Overexpression of the MPO is shown in red.

Top 30 hub proteins were obtained respectively by the Degree and Closeness method in CytoHubba (**Figures 7A, B**). Top 10 hub proteins in two methods and proteins in KEGG pathways were screened (**Table 3**). Finally, one hub protein was identified as the key protein by intersection of KEGG pathways and the two methods, which was Myeloperoxidase (MPO) (**Figure 8**). The expression of MPO protein was relatively high in AS group. Its related KEGG pathway is the Phagosome pathway (**Figure 9**). The upregulation of MPO protein may be involved in the pathogenesis of AS-affected hip joint through the Phagosome pathway.

**Table 3:** DEPs in the KEGG pathways, Degree method, and Closeness method.

Degree method	Closeness method	4 KEGG pathways
VPS35, HIST2H3PS2, H6PD, PTBP1, ELANE, MPO, NUDT21, PKLR, VPS33A, HNRNPH1	VPS35, ARCN1, RPL18A, MPO, SRP54, CDIPT, PKLR, HIST2H3PS2, SEC24D, ELANE	H6PD, RPIA, TKT, PLOD1, PLOD2, PLOD3, SETD7, CDIPT, GPD1, LYPLA2, PHOSPHO1, PLD3, ATP6V1C1, CTSS, DYNC1LI2, ITGB5, MPO, MRC2, TUBB, TUBB2B, TUBB3

### 3.7 Cell Culture and Verification of Inflammation

**Figure 10:**
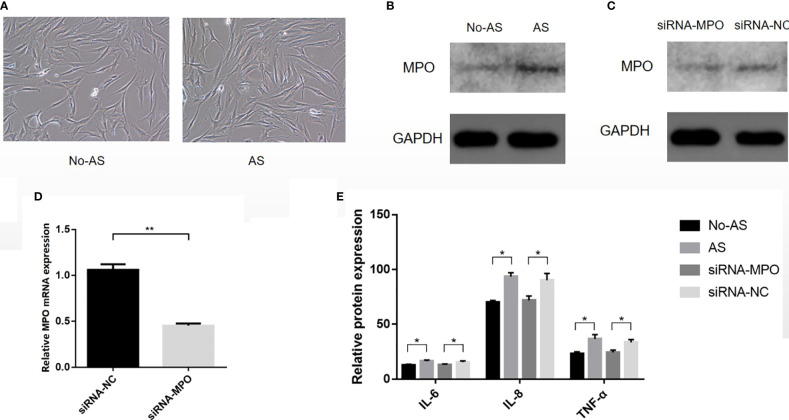
The cells were isolated from the tissue, cultured, and then transfected **(A)**. Western blot analysis showed that the expression of MPO in AS group was higher than that in NO-AS group (P < 0.05) **(B)**. After transfection, the mRNA and protein expressions of MPO in siRNA-MPO group were lower than those in siRNA-NC group (P < 0.05) **(C, D)**. ELISA analysis showed that the expression levels of IL-6, IL-8, and TNF-α in AS group were higher than those in NO-AS group (P < 0.05). The expression levels of IL-6, IL-8, and TNF-α in siRNA-MPO group were lower than those in siRNA-NC group (P < 0.05) **(E)**. *P < 0.05, **P < 0.01.

The results of primary fibroblast culture are shown in **Figure 10A, B**. Western blot analysis showed that the expression of MPO in the AS group was higher than that in the NO-AS group (P < 0.05) (**Figure 10C**). After transfection, the mRNA and protein expressions of MPO in the siRNA-MPO group were lower than those in the siRNA-NC group (P < 0.05) (**Figure 10D**). This suggested that siRNA-MPO treatment of AS fibroblasts resulted in the downregulation of MPO expression. ELISA analysis showed that the expression levels of IL-6, IL-8, and TNF-α in the AS group were higher than those in the NO-AS group (P < 0.05). The expression levels of IL-6, IL-8, and TNF-α in the siRNA-MPO group were lower than those in the siRNA-NC group (P < 0.05). (**Figure 10E**). This suggested that the high expression of MPO in AS cells may promote the high expression of inflammatory factors such AS IL-6, IL-8, and TNF-α. However, after siRNA-MPO treatment, the expression of MPO decreased and resulted in the downregulation of IL-6, IL-8, and TNF-α.

## 4 Discussion

Hip arthropathy is very common in patients with AS. AS with chronic sacroiliac arthritis may lead to severe hip joint lesions, including malformation of the femur head (23, 24). This particular hip lesion prevents many young adults from walking normally and requires hip arthroplasty to improve quality of life (25–27). The molecular mechanism of hip arthropathy in AS has been rarely reported in the past. Therefore, we analyzed the hip ligament tissue between the AS group and the NO-AS group by combining proteomics with bioinformatics, and obtained 193 DEPs. GO functional analysis showed that there were abundant DEPs in BP, CC, and MF. Among them, the number of DEPs enriched in BP and CC was slightly more than that enriched in MF. It was found that MF and BP share common characteristics, including procollagen-lysine 5-dioxygenase activity, peptidyl-lysine 5-dioxygenase activity, and 2-oxoglutarate-dependent dioxygenase activity. MPO was identified as a key protein involved in the phagosome pathway.

In terms of sample collection, we tried our best to control the basic data of the two groups of study population to achieve consistency. However, patients with AS who undergo hip replacement tend to be younger men. This clinical phenomenon is unique and leads to a statistically significant difference in age comparison between AS and non-AS groups. Therefore, we could not control for the difference in age between the two groups, which may be one of the limitations of the study. It was found that there was a high degree of consistency between the two groups of ligament samples, indicating that the collection and preservation of ligament samples was successful. This provides a preliminary guarantee for the accuracy of subsequent experiments. Although DEPs slightly tended to enrich in BP and CC, the difference was not obvious. In the PPI network, two different algorithms are calculated by Degree method and closeness method, which may be conducive to improving the accuracy of key proteins. Finally, MPO related to Phagosome pathway was screened out from DEPs related to KEGG pathway. The upregulated expression of MPO in the Phagosome pathway may be a mechanism of AS hip lesions.

MPO is a member of the heme-containing peroxidase subfamily. It is usually expressed in large amounts in various immune cells such as neutrophils, lymphocytes, monocytes, macrophages, and so on (28–32). MPO is usually stored in eosinophilic particles inside the cell. When pathogenic bacteria invade the body, these particles will enter the extracellular space through degranulation or exocrine (28, 29). Neutrophils are a line of defense against pathogens. When the pathogen enters the tissue, the neutrophils engulf the pathogen and place it on the phagosome. Most pathogens are killed and digested in the phagosomes (33, 34). In neutrophils, eosinophilic granules containing MPO fuse with the phagosome. When the endoplasmic membrane is ruptured due to pathogen invasion, substances in particles including MPO will be released (35, 36). Inequilibrium of cellular degranulation mechanisms is a common feature of many inflammatory diseases, such as acute lung injury, rheumatoid arthritis, psoriasis, etc. (37–39). However, the mechanism controlling cell degranulation is not clear. Some studies suggest that cellular degranulation may depend on the activation of intracellular signaling pathways, including the Src family of tyrosine kinases, β-arrestin, et al. (36, 40).

The neutrophil degranulation results in an increase in MPO protein concentration. MPO catalyzes various substrates and disproportionates superoxides. Under the condition of sufficient chlorine, H2O2 is catalyzed to effectively convert to HOCl (41, 42). Klebanoff SJ (43) found that the antimicrobial system formed in the phagosomes included MPO, H2O2, and halides (especially chlorides). The initial product of MPO-H2O2-Cl system is HOCl, followed by the formation of chlorine, chloramine, hydroxyl radical, singlet oxygen, and ozone. These toxic substances can be released outside the cell, where they may attack normal tissue and trigger disease. Green JN et al. (44) believe that HOCl can react with granulosin and produce 3-chlorotyrosine, protein carbonyl group, and a large amount of chloramines. HOCl may also kill ingested bacteria through indirect mechanisms involving protein oxidation and monochloramine formation. Odobasic D et al. (33) pointed out that substances generated by the reaction include HOCl, hypothiocyanate, tyrosine free radicals and active nitrogen intermediates, etc., which can have a profound impact on cell functions by modifying proteins, lipids, and DNA. Mayadas TN et al. (45) found that neutrophils play an important role as effector cells in many inflammatory and autoimmune diseases through phagocytosis, release of reactive oxygen species, degranulation, extracellular trap, proinflammatory cytokines, and protease mediation. These diseases include atherosclerosis, cardiovascular disease, inflammatory diseases of the lungs and kidneys, and rheumatoid arthritis. Van der Veen BS et al. (46) also pointed out that excessive production of MPO-derived oxidants is associated with tissue damage in many diseases, especially those characterized by acute or chronic inflammation. MPO may play a role beyond its oxidative properties, which may be independent of its catalytic activity and affect various processes of cellular signaling and cell-cell interactions, thus regulating the inflammatory response. Pitanga TN et al. (47) believe that the corpuscule-associated MPO-H2O2-Cl system may be involved in extensive endothelial cell injury under the condition of neutrophil activation. Panagopoulos V et al. (48) found that MPO inhibits the differentiation of osteoclasts in inflammatory sites, participates in the formation of extracellular matrix, angiogenesis, and bone mineralization, and plays an important role in bone integrity. Past studies have shown that MPO is an active disease biomarker associated with a variety of diseases, such as cardiovascular disease, cancer, kidney disease, lung injury, rheumatoid arthritis, and multiple sclerosis (28, 29, 49–51). MPO is an important participant in inflammation and immune response in the body. It has a variety of functions in tissues, such as participating in sterile inflammatory response, maintaining normal immune response, remodeling local microvessels, and enhancing the activity of osteoblasts and osteoclasts. Abnormal expression of MPO can lead to a range of diseases. The phagocytes are generally considered to be important structures in the processing of antigens by various immune cells. The recognition and processing of exogenous antigens by neutrophils and macrophages is an important part of the non-specific immune response (52). Normal patients have no exogenous antigens in the hip joint. In AS-affected hip patients, overexpression of MPO may result in abnormal cell activity, including immune cells. These abnormalities activate the phagosome pathway and treat the tissue as an antigen, inducing aseptic inflammatory response and autoimmune response. **Figure 9** shows the role of MPO in the phagosome pathway. The phagosomes contain MPO, which catalyzes H2O2 and produces HClO. They participate in antigen processing and produce a series of cellular effects. In our study, MPO overexpression may be one of the factors leading to aseptic inflammatory and immune responses in the hip joint.

AS is considered as a chronic inflammatory autoimmune disease, which can cause spinal stiffness and fibrosis, as well as various degrees of damage to muscles, bones, lungs, eyes, and other parts. Ligament attachment site is one of the main pathological sites of AS. And fibroblasts are the main components of ligaments. Abnormal expression of MPO may contribute to changes in the physiological activity of fibroblasts. On the basis of cell culture, we explored whether upregulated MPO led to the inflammatory response of AS fibroblasts by interfering with the overexpression of MPO. As a result, we further confirmed that MPO was upregulated in AS hip fibroblasts. By inhibiting the overexpression of MPO, the expression of inflammatory factors such AS IL-6, IL-8, and TNF-α in AS hip fibroblasts could be downregulated. Therefore, overexpression of MPO may induce upregulation of inflammatory cytokines such as IL-6, IL-8, and TNF-α in fibroblasts. This can be one of the factors that contribute to the autoinflammatory response of the AS-diseased hip joint.

The mechanism of MPO synthesis has not been fully elucidated (53). The gene encoding human MPO may contain 12 exons and 11 introns, located on chromosome 17q22 (54). Lin Km et al. (55) indicated that there are three different MPO promoters in the 5’ flanking region of human MPO gene, and they may regulate the transcription of MPO. Zhao WG et al. (56) believed that only one of the three identified promoter regions of human MPO was active. But all three played a role in the expression of MPO in mice. Some studies indicate that MPO biosynthesis begins with PreMPO, which is a primary translated product produced in the endoplasmic reticulum. The PreMPO is processed to form apoproMPO. ApoPrompo binds returnably with the molecular chaperone and obtains heme to become the enzymatic active ProMPO. After leaving the endoplasmic reticulum, proMPO enters the secretion pathway, most of which are formed into mature MPO through proteolysis and dimerization, and a small part is secreted out of the cell in the form of single proMPO (30, 57). The synthesis and processing of MPO is very complex. Many factors are interrelated and play a controlling role in the expression of MPO in an organization. Nauseef WM (30) suggested that there is a need to explore the possibility that nutritional factors produced in a specific inflammatory environment may promote the revival of MPO gene expression in cells that do not normally produce MPO. Nagra RM et al. (51) believed that macrophages do not actively produce MPO, but MPO is present in microglia/macrophages in and around the lesion of multiple sclerosis. MPO is closely associated with inflammatory response and may promote the production of inflammatory cytokines through multiple pathways (28, 29, 31). In the experiment, the overexpression of MPO and inflammatory cytokines (IL-6, IL-8, TNF-α) in fibroblasts may indicate that fibroblasts are present in the posture of inflammatory response.

## 5 Conclusion

The mechanism of hip arthropathy in AS has been explored by combining proteomics and bioinformatics. It was found that abnormal MPO may influence AS hip lesions through the phagosome pathway. Overexpression of MPO may induce aseptic inflammatory response and autoimmune response in AS-diseased hip joint. This discovery could facilitate future molecular-targeted therapies for AS and prevent hip disease.

## Future Perspective

AS is associated with hip joint lesions, which seriously affects the patient’s quality of life and requires surgical treatment. In the past, the overall pathogenesis of AS has not been elucidate, and the mechanism of hip arthropathy in AS has rarely been studied. We screened for potential protein biomarkers leading to hip arthropathy in AS by a combination of label-free quantification analysis (LFQ) and bioinformatics. The key proteins were verified by a series of experiments. This study provides a preliminary understanding of the molecular characteristics of hip arthropathy in AS, contributes to a further comprehensive understanding of the biological characteristics of AS, and provides a new molecular target for personalized treatment.

## Summary Points

- We performed a proteomic analysis of diseased hip in AS by a marker-free quantification (LFQ) technique.
- We used bioinformatics techniques to screen key differentially expressed proteins (myeloperoxidase) and KEGG pathways (phagosome).
- In a series of experiments, we further demonstrated that overexpression of MPO promoted the inflammatory response of fibroblasts in AS-diseased hip joints.
- Our study provides new insights into the pathogenesis of AS hip joint and provides new molecular targets for personalized therapy.

## Data Availability Statement

The datasets presented in this study can be found in online repositories. The names of the repository/repositories and accession number(s) can be found in the article/Supplementary Material .

## Ethics Statement

The studies involving human participants were reviewed and approved by the First Affiliated Hospital of Guangxi Medical University. The patients/participants provided their written informed consent to participate in this study.

## Author Contributions

CY designed the study. XZ and CL supervised the study. CY, TL, CL, ZZ, JJ, JX, and JC analyzed the data. CY and ZZ performed digital visualization. CY wrote and revised the manuscript. All authors contributed to the article and approved the submitted version.

## Funding

This work was supported by National Natural Science Foundation of China (No. 81560359 and 81860393).

## Conflict of Interest

The authors declare that the research was conducted in the absence of any commercial or financial relationships that could be construed as a potential conflict of interest.

## Publisher’s Note

All claims expressed in this article are solely those of the authors and do not necessarily represent those of their affiliated organizations, or those of the publisher, the editors and the reviewers. Any product that may be evaluated in this article, or claim that may be made by its manufacturer, is not guaranteed or endorsed by the publisher.
